# Supplementary material for: Modeling glioblastoma heterogeneity as a dynamic network of cell states
Source: Mol Syst Biol. 2021 Sep 16;17(9):e10105. doi: 10.15252/msb.202010105 (PMC8444284; doi:10.15252/msb.202010105)
Supplement: Supplementary file 6 — Source Data for Figure 5 [file MSB-17-e10105-s004.zip › Figure5A_sourcedata/GSEA_3071/nosigdb_stateI.GseaPreranked.1622795145664/RICHARDS_NOWAKOWSKI_SCIENCE_2017_IPC_DIV1_UPREG.html]

Details for gene set RICHARDS\_NOWAKOWSKI\_SCIENCE\_2017\_IPC\_DIV1\_UPREG[GSEA]

|  || Dataset | state13071 |
| Phenotype | NoPhenotypeAvailable |
| Upregulated in class | na\_neg |
| GeneSet | RICHARDS\_NOWAKOWSKI\_SCIENCE\_2017\_IPC\_DIV1\_UPREG |
| Enrichment Score (ES) | -0.4769965 |
| Normalized Enrichment Score (NES) | -3.0012424 |
| Nominal p-value | 0.0 |
| FDR q-value | 0.0 |
| FWER p-Value | 0.0 |
Table: GSEA Results Summary

  

Fig 1: Enrichment plot: RICHARDS\_NOWAKOWSKI\_SCIENCE\_2017\_IPC\_DIV1\_UPREG      
 Profile of the Running ES Score & Positions of GeneSet Members on the Rank Ordered List

  

| PROBE | GENE SYMBOL | GENE\_TITLE | RANK IN GENE LIST | RANK METRIC SCORE | RUNNING ES | CORE ENRICHMENT || 1 | SLC38A2 |  |  | 200 | 0.826 | -0.0611 | No |
| 2 | LMO7 |  |  | 206 | 0.813 | -0.0592 | No |
| 3 | STK17B |  |  | 274 | 0.707 | -0.0778 | No |
| 4 | PHGDH |  |  | 279 | 0.699 | -0.0761 | No |
| 5 | KIAA0232 |  |  | 293 | 0.685 | -0.0774 | No |
| 6 | CLMP |  |  | 307 | 0.665 | -0.0787 | No |
| 7 | ZMIZ1 |  |  | 365 | 0.624 | -0.0945 | No |
| 8 | MAGED1 |  |  | 391 | 0.599 | -0.1000 | No |
| 9 | ANXA5 |  |  | 392 | 0.597 | -0.0974 | No |
| 10 | PRDX1 |  |  | 447 | 0.549 | -0.1125 | No |
| 11 | BASP1 |  |  | 457 | 0.544 | -0.1131 | No |
| 12 | TANK |  |  | 464 | 0.538 | -0.1128 | No |
| 13 | FSTL1 |  |  | 505 | 0.504 | -0.1235 | No |
| 14 | SEC11A |  |  | 518 | 0.496 | -0.1253 | No |
| 15 | LAPTM4A |  |  | 553 | 0.478 | -0.1342 | No |
| 16 | CD63 |  |  | 618 | 0.444 | -0.1530 | No |
| 17 | OS9 |  |  | 693 | 0.414 | -0.1751 | No |
| 18 | SMIM14 |  |  | 725 | 0.403 | -0.1834 | No |
| 19 | PIP4K2A |  |  | 736 | 0.401 | -0.1849 | No |
| 20 | HSD17B11 |  |  | 779 | 0.389 | -0.1968 | No |
| 21 | HSP90B1 |  |  | 798 | 0.382 | -0.2010 | No |
| 22 | CLIC4 |  |  | 800 | 0.382 | -0.1997 | No |
| 23 | LRRC8A |  |  | 815 | 0.376 | -0.2026 | No |
| 24 | HMGCR |  |  | 875 | 0.357 | -0.2201 | No |
| 25 | SGCE |  |  | 891 | 0.353 | -0.2235 | No |
| 26 | DENND1B |  |  | 1041 | 0.320 | -0.2702 | No |
| 27 | UBXN4 |  |  | 1234 | 0.282 | -0.3310 | No |
| 28 | ADD3 |  |  | 1261 | 0.275 | -0.3382 | No |
| 29 | PHLDA1 |  |  | 1262 | 0.275 | -0.3370 | No |
| 30 | CTNND1 |  |  | 1338 | 0.261 | -0.3601 | No |
| 31 | CCDC34 |  |  | 1376 | 0.255 | -0.3710 | No |
| 32 | TSHZ1 |  |  | 1377 | 0.255 | -0.3699 | No |
| 33 | VPS36 |  |  | 1408 | 0.251 | -0.3785 | No |
| 34 | CEP152 |  |  | 1446 | -0.253 | -0.3894 | No |
| 35 | CTCF |  |  | 1450 | -0.253 | -0.3893 | No |
| 36 | GAPDH |  |  | 1459 | -0.253 | -0.3908 | No |
| 37 | PLGRKT |  |  | 1466 | -0.254 | -0.3916 | No |
| 38 | GTF2I |  |  | 1476 | -0.255 | -0.3934 | No |
| 39 | SRSF10 |  |  | 1489 | -0.256 | -0.3962 | No |
| 40 | ZGRF1 |  |  | 1505 | -0.258 | -0.4000 | No |
| 41 | PHC2 |  |  | 1521 | -0.259 | -0.4037 | No |
| 42 | USP11 |  |  | 1522 | -0.260 | -0.4026 | No |
| 43 | C4orf46 |  |  | 1596 | -0.267 | -0.4250 | No |
| 44 | AGO1 |  |  | 1606 | -0.268 | -0.4268 | No |
| 45 | CCNG2 |  |  | 1610 | -0.269 | -0.4266 | No |
| 46 | C2orf69 |  |  | 1617 | -0.269 | -0.4274 | No |
| 47 | TTK |  |  | 1620 | -0.270 | -0.4269 | No |
| 48 | MPHOSPH9 |  |  | 1623 | -0.270 | -0.4264 | No |
| 49 | SSH2 |  |  | 1637 | -0.272 | -0.4294 | No |
| 50 | SAE1 |  |  | 1638 | -0.273 | -0.4283 | No |
| 51 | SUMO2 |  |  | 1643 | -0.273 | -0.4284 | No |
| 52 | SMC3 |  |  | 1650 | -0.274 | -0.4291 | No |
| 53 | GADD45G |  |  | 1657 | -0.275 | -0.4299 | No |
| 54 | KIAA1191 |  |  | 1661 | -0.275 | -0.4297 | No |
| 55 | TBC1D1 |  |  | 1674 | -0.277 | -0.4324 | No |
| 56 | SNCAIP |  |  | 1686 | -0.278 | -0.4348 | No |
| 57 | BUB3 |  |  | 1687 | -0.278 | -0.4336 | No |
| 58 | YBX1 |  |  | 1725 | -0.283 | -0.4443 | No |
| 59 | PNRC2 |  |  | 1731 | -0.284 | -0.4447 | No |
| 60 | KIF2C |  |  | 1735 | -0.284 | -0.4445 | No |
| 61 | NEUROD1 |  |  | 1751 | -0.287 | -0.4481 | No |
| 62 | PSMC3 |  |  | 1752 | -0.287 | -0.4469 | No |
| 63 | MIDN |  |  | 1755 | -0.287 | -0.4463 | No |
| 64 | CCNB2 |  |  | 1757 | -0.288 | -0.4454 | No |
| 65 | ZNF850 |  |  | 1759 | -0.288 | -0.4445 | No |
| 66 | ZNF273 |  |  | 1760 | -0.288 | -0.4432 | No |
| 67 | CALM2 |  |  | 1763 | -0.289 | -0.4426 | No |
| 68 | MIS18BP1 |  |  | 1764 | -0.289 | -0.4414 | No |
| 69 | YAF2 |  |  | 1766 | -0.290 | -0.4405 | No |
| 70 | CENPJ |  |  | 1768 | -0.290 | -0.4396 | No |
| 71 | EXOSC9 |  |  | 1777 | -0.291 | -0.4409 | No |
| 72 | PPP2R5C |  |  | 1780 | -0.291 | -0.4403 | No |
| 73 | APOLD1 |  |  | 1781 | -0.291 | -0.4391 | No |
| 74 | TRIM28 |  |  | 1789 | -0.292 | -0.4401 | No |
| 75 | GKAP1 |  |  | 1792 | -0.293 | -0.4395 | No |
| 76 | PIK3R3 |  |  | 1797 | -0.293 | -0.4395 | No |
| 77 | TRIM2 |  |  | 1803 | -0.294 | -0.4399 | No |
| 78 | DANCR |  |  | 1807 | -0.294 | -0.4396 | No |
| 79 | HNRNPA2B1 |  |  | 1819 | -0.296 | -0.4419 | No |
| 80 | TROAP |  |  | 1821 | -0.296 | -0.4409 | No |
| 81 | RUVBL2 |  |  | 1829 | -0.297 | -0.4419 | No |
| 82 | ARPP19 |  |  | 1837 | -0.298 | -0.4429 | No |
| 83 | MSANTD3 |  |  | 1842 | -0.298 | -0.4429 | No |
| 84 | RHOA |  |  | 1843 | -0.298 | -0.4417 | No |
| 85 | CDON |  |  | 1852 | -0.300 | -0.4430 | No |
| 86 | FOXN2 |  |  | 1854 | -0.300 | -0.4420 | No |
| 87 | CENPQ |  |  | 1860 | -0.300 | -0.4423 | No |
| 88 | LSM12 |  |  | 1870 | -0.301 | -0.4440 | No |
| 89 | TTF2 |  |  | 1873 | -0.301 | -0.4433 | No |
| 90 | MSI1 |  |  | 1888 | -0.303 | -0.4466 | No |
| 91 | RNF114 |  |  | 1899 | -0.305 | -0.4485 | No |
| 92 | MDM2 |  |  | 1904 | -0.305 | -0.4485 | No |
| 93 | RAN |  |  | 1907 | -0.306 | -0.4478 | No |
| 94 | TACC1 |  |  | 1916 | -0.307 | -0.4491 | No |
| 95 | POLR2E |  |  | 1941 | -0.312 | -0.4555 | No |
| 96 | KPNA2 |  |  | 1944 | -0.312 | -0.4548 | No |
| 97 | COPS6 |  |  | 1949 | -0.312 | -0.4548 | No |
| 98 | SRR |  |  | 1950 | -0.312 | -0.4534 | No |
| 99 | EXOG |  |  | 1952 | -0.313 | -0.4524 | No |
| 100 | TGIF1 |  |  | 1955 | -0.313 | -0.4517 | No |
| 101 | DCAF7 |  |  | 1961 | -0.313 | -0.4520 | No |
| 102 | ZNF92 |  |  | 1962 | -0.313 | -0.4507 | No |
| 103 | ARMC1 |  |  | 1963 | -0.313 | -0.4493 | No |
| 104 | SPDL1 |  |  | 1968 | -0.314 | -0.4493 | No |
| 105 | VEZF1 |  |  | 1970 | -0.314 | -0.4483 | No |
| 106 | VBP1 |  |  | 1993 | -0.317 | -0.4540 | No |
| 107 | CEP78 |  |  | 2000 | -0.317 | -0.4546 | No |
| 108 | SUZ12 |  |  | 2011 | -0.318 | -0.4565 | No |
| 109 | SMARCD1 |  |  | 2016 | -0.319 | -0.4564 | No |
| 110 | INCENP |  |  | 2018 | -0.320 | -0.4553 | No |
| 111 | PARPBP |  |  | 2026 | -0.322 | -0.4562 | No |
| 112 | STT3B |  |  | 2029 | -0.322 | -0.4555 | No |
| 113 | AMOTL2 |  |  | 2031 | -0.323 | -0.4544 | No |
| 114 | EFS |  |  | 2038 | -0.323 | -0.4550 | No |
| 115 | SUV39H2 |  |  | 2041 | -0.324 | -0.4543 | No |
| 116 | DDX39A |  |  | 2043 | -0.324 | -0.4532 | No |
| 117 | DNA2 |  |  | 2057 | -0.327 | -0.4560 | No |
| 118 | TDP1 |  |  | 2059 | -0.327 | -0.4549 | No |
| 119 | SNRPB |  |  | 2064 | -0.328 | -0.4548 | No |
| 120 | MZT2B |  |  | 2075 | -0.330 | -0.4566 | No |
| 121 | SRRT |  |  | 2077 | -0.331 | -0.4556 | No |
| 122 | E2F7 |  |  | 2081 | -0.331 | -0.4551 | No |
| 123 | ZNF93 |  |  | 2095 | -0.333 | -0.4579 | No |
| 124 | CBX1 |  |  | 2096 | -0.334 | -0.4565 | No |
| 125 | C19orf53 |  |  | 2099 | -0.334 | -0.4557 | No |
| 126 | CHEK1 |  |  | 2108 | -0.336 | -0.4568 | No |
| 127 | NDE1 |  |  | 2111 | -0.337 | -0.4560 | No |
| 128 | FDFT1 |  |  | 2117 | -0.337 | -0.4562 | No |
| 129 | EZR |  |  | 2123 | -0.338 | -0.4564 | No |
| 130 | C18orf54 |  |  | 2128 | -0.339 | -0.4562 | No |
| 131 | CORO1C |  |  | 2136 | -0.340 | -0.4570 | No |
| 132 | TIMM8B |  |  | 2139 | -0.341 | -0.4562 | No |
| 133 | PSPC1 |  |  | 2140 | -0.341 | -0.4548 | No |
| 134 | TMEM256 |  |  | 2150 | -0.343 | -0.4562 | No |
| 135 | PHIP |  |  | 2162 | -0.345 | -0.4583 | No |
| 136 | PLK4 |  |  | 2167 | -0.346 | -0.4581 | No |
| 137 | KIAA1958 |  |  | 2170 | -0.347 | -0.4572 | No |
| 138 | TNPO2 |  |  | 2180 | -0.348 | -0.4587 | No |
| 139 | ZNF85 |  |  | 2185 | -0.349 | -0.4585 | No |
| 140 | HNRNPL |  |  | 2190 | -0.350 | -0.4583 | No |
| 141 | DLEU2 |  |  | 2196 | -0.351 | -0.4584 | No |
| 142 | FAM83D |  |  | 2198 | -0.351 | -0.4572 | No |
| 143 | POC1A |  |  | 2199 | -0.351 | -0.4557 | No |
| 144 | PLK1 |  |  | 2206 | -0.353 | -0.4561 | No |
| 145 | RANGAP1 |  |  | 2207 | -0.354 | -0.4546 | No |
| 146 | CENPA |  |  | 2220 | -0.357 | -0.4570 | No |
| 147 | SOGA1 |  |  | 2234 | -0.360 | -0.4596 | No |
| 148 | POLQ |  |  | 2253 | -0.364 | -0.4639 | No |
| 149 | DZIP3 |  |  | 2260 | -0.365 | -0.4643 | No |
| 150 | NUCKS1 |  |  | 2278 | -0.368 | -0.4682 | No |
| 151 | OTUD6B |  |  | 2284 | -0.369 | -0.4682 | No |
| 152 | DEPDC1B |  |  | 2288 | -0.370 | -0.4676 | No |
| 153 | TUBB4B |  |  | 2291 | -0.370 | -0.4667 | No |
| 154 | FUS |  |  | 2300 | -0.373 | -0.4677 | No |
| 155 | CEP128 |  |  | 2323 | -0.377 | -0.4732 | No |
| 156 | CKS1B |  |  | 2332 | -0.380 | -0.4741 | No |
| 157 | BUB1B |  |  | 2335 | -0.381 | -0.4731 | No |
| 158 | ING1 |  |  | 2337 | -0.382 | -0.4718 | No |
| 159 | NUP50 |  |  | 2354 | -0.385 | -0.4754 | Yes |
| 160 | CKAP2 |  |  | 2357 | -0.385 | -0.4744 | Yes |
| 161 | PSIP1 |  |  | 2364 | -0.386 | -0.4746 | Yes |
| 162 | DKC1 |  |  | 2365 | -0.386 | -0.4730 | Yes |
| 163 | CCNF |  |  | 2370 | -0.387 | -0.4726 | Yes |
| 164 | STIL |  |  | 2374 | -0.388 | -0.4719 | Yes |
| 165 | H3F3A |  |  | 2377 | -0.388 | -0.4709 | Yes |
| 166 | RBMX |  |  | 2378 | -0.388 | -0.4693 | Yes |
| 167 | HAUS6 |  |  | 2388 | -0.390 | -0.4705 | Yes |
| 168 | CENPW |  |  | 2402 | -0.393 | -0.4730 | Yes |
| 169 | UHRF1 |  |  | 2404 | -0.393 | -0.4717 | Yes |
| 170 | PRIM2 |  |  | 2409 | -0.394 | -0.4713 | Yes |
| 171 | NEIL3 |  |  | 2413 | -0.395 | -0.4706 | Yes |
| 172 | BUB1 |  |  | 2416 | -0.396 | -0.4695 | Yes |
| 173 | STRBP |  |  | 2420 | -0.397 | -0.4688 | Yes |
| 174 | WEE1 |  |  | 2426 | -0.397 | -0.4687 | Yes |
| 175 | H2AFV |  |  | 2431 | -0.398 | -0.4683 | Yes |
| 176 | KCTD9 |  |  | 2438 | -0.399 | -0.4685 | Yes |
| 177 | EMC9 |  |  | 2445 | -0.400 | -0.4688 | Yes |
| 178 | HNRNPA1 |  |  | 2455 | -0.402 | -0.4700 | Yes |
| 179 | CENPO |  |  | 2460 | -0.404 | -0.4695 | Yes |
| 180 | CKS2 |  |  | 2466 | -0.404 | -0.4694 | Yes |
| 181 | AURKA |  |  | 2467 | -0.404 | -0.4677 | Yes |
| 182 | BLM |  |  | 2469 | -0.404 | -0.4663 | Yes |
| 183 | TGIF2 |  |  | 2474 | -0.406 | -0.4658 | Yes |
| 184 | KIF11 |  |  | 2488 | -0.410 | -0.4683 | Yes |
| 185 | HAUS8 |  |  | 2494 | -0.412 | -0.4681 | Yes |
| 186 | CENPL |  |  | 2497 | -0.413 | -0.4670 | Yes |
| 187 | UCP2 |  |  | 2498 | -0.413 | -0.4653 | Yes |
| 188 | POLR2D |  |  | 2510 | -0.416 | -0.4670 | Yes |
| 189 | SPAG5 |  |  | 2519 | -0.418 | -0.4678 | Yes |
| 190 | RACGAP1 |  |  | 2522 | -0.419 | -0.4667 | Yes |
| 191 | CDC20 |  |  | 2540 | -0.425 | -0.4704 | Yes |
| 192 | PARP2 |  |  | 2542 | -0.425 | -0.4689 | Yes |
| 193 | FIGN |  |  | 2543 | -0.426 | -0.4670 | Yes |
| 194 | MEX3A |  |  | 2559 | -0.429 | -0.4701 | Yes |
| 195 | NCAPD2 |  |  | 2563 | -0.431 | -0.4692 | Yes |
| 196 | CDK5RAP2 |  |  | 2564 | -0.431 | -0.4673 | Yes |
| 197 | ZNF100 |  |  | 2567 | -0.431 | -0.4661 | Yes |
| 198 | RSRC1 |  |  | 2571 | -0.433 | -0.4653 | Yes |
| 199 | H2AFZ |  |  | 2572 | -0.433 | -0.4634 | Yes |
| 200 | ATCAY |  |  | 2583 | -0.435 | -0.4648 | Yes |
| 201 | PPP4R2 |  |  | 2586 | -0.436 | -0.4636 | Yes |
| 202 | ANP32B |  |  | 2593 | -0.438 | -0.4636 | Yes |
| 203 | CIT |  |  | 2604 | -0.440 | -0.4650 | Yes |
| 204 | KNTC1 |  |  | 2613 | -0.443 | -0.4657 | Yes |
| 205 | NCAPG2 |  |  | 2621 | -0.445 | -0.4660 | Yes |
| 206 | FANCB |  |  | 2623 | -0.446 | -0.4645 | Yes |
| 207 | SRSF3 |  |  | 2631 | -0.447 | -0.4648 | Yes |
| 208 | HIRIP3 |  |  | 2635 | -0.449 | -0.4638 | Yes |
| 209 | LINC01224 |  |  | 2637 | -0.449 | -0.4623 | Yes |
| 210 | DCTPP1 |  |  | 2644 | -0.451 | -0.4623 | Yes |
| 211 | MASTL |  |  | 2646 | -0.452 | -0.4607 | Yes |
| 212 | SUGP2 |  |  | 2650 | -0.454 | -0.4597 | Yes |
| 213 | EXOSC8 |  |  | 2659 | -0.456 | -0.4603 | Yes |
| 214 | CCDC152 |  |  | 2664 | -0.460 | -0.4596 | Yes |
| 215 | CCNA2 |  |  | 2667 | -0.462 | -0.4583 | Yes |
| 216 | RFWD3 |  |  | 2668 | -0.462 | -0.4563 | Yes |
| 217 | SKA2 |  |  | 2672 | -0.464 | -0.4553 | Yes |
| 218 | MND1 |  |  | 2682 | -0.468 | -0.4562 | Yes |
| 219 | KIF22 |  |  | 2683 | -0.468 | -0.4542 | Yes |
| 220 | PAX6 |  |  | 2688 | -0.469 | -0.4535 | Yes |
| 221 | CMC2 |  |  | 2694 | -0.470 | -0.4531 | Yes |
| 222 | CDCA8 |  |  | 2695 | -0.470 | -0.4511 | Yes |
| 223 | MMS22L |  |  | 2698 | -0.471 | -0.4498 | Yes |
| 224 | ANLN |  |  | 2702 | -0.472 | -0.4487 | Yes |
| 225 | PRC1 |  |  | 2705 | -0.472 | -0.4473 | Yes |
| 226 | FANCD2 |  |  | 2707 | -0.474 | -0.4456 | Yes |
| 227 | CENPN |  |  | 2708 | -0.474 | -0.4436 | Yes |
| 228 | MELK |  |  | 2709 | -0.474 | -0.4416 | Yes |
| 229 | HEG1 |  |  | 2714 | -0.475 | -0.4409 | Yes |
| 230 | TUBG1 |  |  | 2715 | -0.475 | -0.4388 | Yes |
| 231 | PHLPP1 |  |  | 2717 | -0.475 | -0.4371 | Yes |
| 232 | KNSTRN |  |  | 2722 | -0.478 | -0.4364 | Yes |
| 233 | NDC80 |  |  | 2723 | -0.478 | -0.4343 | Yes |
| 234 | SHCBP1 |  |  | 2733 | -0.480 | -0.4352 | Yes |
| 235 | BRIP1 |  |  | 2739 | -0.482 | -0.4347 | Yes |
| 236 | TIMELESS |  |  | 2752 | -0.487 | -0.4365 | Yes |
| 237 | CENPF |  |  | 2761 | -0.490 | -0.4370 | Yes |
| 238 | ACTL6A |  |  | 2762 | -0.492 | -0.4349 | Yes |
| 239 | SLC25A5 |  |  | 2765 | -0.493 | -0.4335 | Yes |
| 240 | BRCA2 |  |  | 2772 | -0.495 | -0.4333 | Yes |
| 241 | MRPL47 |  |  | 2773 | -0.495 | -0.4312 | Yes |
| 242 | ENC1 |  |  | 2776 | -0.496 | -0.4297 | Yes |
| 243 | NUF2 |  |  | 2781 | -0.497 | -0.4289 | Yes |
| 244 | JAM2 |  |  | 2784 | -0.498 | -0.4274 | Yes |
| 245 | TUBA1B |  |  | 2785 | -0.498 | -0.4252 | Yes |
| 246 | COX17 |  |  | 2786 | -0.498 | -0.4231 | Yes |
| 247 | SKA1 |  |  | 2787 | -0.498 | -0.4210 | Yes |
| 248 | CHRDL1 |  |  | 2793 | -0.499 | -0.4205 | Yes |
| 249 | DTYMK |  |  | 2798 | -0.501 | -0.4196 | Yes |
| 250 | WDR76 |  |  | 2800 | -0.501 | -0.4178 | Yes |
| 251 | DCAF16 |  |  | 2801 | -0.501 | -0.4157 | Yes |
| 252 | RAD18 |  |  | 2802 | -0.501 | -0.4135 | Yes |
| 253 | HMGB1 |  |  | 2806 | -0.503 | -0.4123 | Yes |
| 254 | USP13 |  |  | 2817 | -0.508 | -0.4134 | Yes |
| 255 | COX8A |  |  | 2819 | -0.509 | -0.4115 | Yes |
| 256 | LSM5 |  |  | 2820 | -0.509 | -0.4094 | Yes |
| 257 | ARL6IP1 |  |  | 2823 | -0.510 | -0.4078 | Yes |
| 258 | CDCA3 |  |  | 2829 | -0.513 | -0.4073 | Yes |
| 259 | TFDP2 |  |  | 2831 | -0.514 | -0.4054 | Yes |
| 260 | SPC24 |  |  | 2836 | -0.514 | -0.4045 | Yes |
| 261 | HMGB3 |  |  | 2839 | -0.516 | -0.4029 | Yes |
| 262 | TOP2A |  |  | 2842 | -0.517 | -0.4014 | Yes |
| 263 | ZNF43 |  |  | 2844 | -0.517 | -0.3995 | Yes |
| 264 | ZBTB18 |  |  | 2850 | -0.521 | -0.3989 | Yes |
| 265 | CEP55 |  |  | 2852 | -0.522 | -0.3970 | Yes |
| 266 | CEP97 |  |  | 2856 | -0.522 | -0.3957 | Yes |
| 267 | CKAP2L |  |  | 2857 | -0.523 | -0.3935 | Yes |
| 268 | ECT2 |  |  | 2858 | -0.523 | -0.3912 | Yes |
| 269 | NUDT1 |  |  | 2859 | -0.524 | -0.3890 | Yes |
| 270 | RFC5 |  |  | 2862 | -0.526 | -0.3874 | Yes |
| 271 | RAD21 |  |  | 2866 | -0.527 | -0.3861 | Yes |
| 272 | HJURP |  |  | 2869 | -0.527 | -0.3845 | Yes |
| 273 | ANP32E |  |  | 2877 | -0.530 | -0.3845 | Yes |
| 274 | PTTG1 |  |  | 2895 | -0.540 | -0.3877 | Yes |
| 275 | TMEM158 |  |  | 2900 | -0.543 | -0.3867 | Yes |
| 276 | RHNO1 |  |  | 2902 | -0.544 | -0.3847 | Yes |
| 277 | ZNF682 |  |  | 2906 | -0.545 | -0.3833 | Yes |
| 278 | HNRNPUL1 |  |  | 2913 | -0.549 | -0.3829 | Yes |
| 279 | FAM111A |  |  | 2917 | -0.552 | -0.3815 | Yes |
| 280 | ARHGAP11A |  |  | 2921 | -0.556 | -0.3801 | Yes |
| 281 | ELAVL3 |  |  | 2924 | -0.559 | -0.3783 | Yes |
| 282 | CCDC14 |  |  | 2929 | -0.560 | -0.3772 | Yes |
| 283 | ATAD5 |  |  | 2935 | -0.563 | -0.3765 | Yes |
| 284 | H2AFY |  |  | 2942 | -0.568 | -0.3760 | Yes |
| 285 | PSRC1 |  |  | 2944 | -0.569 | -0.3739 | Yes |
| 286 | MIS18A |  |  | 2947 | -0.570 | -0.3721 | Yes |
| 287 | KIF4A |  |  | 2953 | -0.574 | -0.3712 | Yes |
| 288 | BLOC1S1 |  |  | 2954 | -0.574 | -0.3688 | Yes |
| 289 | TACC3 |  |  | 2956 | -0.575 | -0.3666 | Yes |
| 290 | KIF23 |  |  | 2961 | -0.579 | -0.3655 | Yes |
| 291 | MZT1 |  |  | 2964 | -0.580 | -0.3636 | Yes |
| 292 | NF2 |  |  | 2972 | -0.583 | -0.3634 | Yes |
| 293 | NCAPH |  |  | 2974 | -0.585 | -0.3612 | Yes |
| 294 | ZNF714 |  |  | 2977 | -0.586 | -0.3594 | Yes |
| 295 | VRK1 |  |  | 2983 | -0.592 | -0.3585 | Yes |
| 296 | SMC2 |  |  | 2984 | -0.592 | -0.3559 | Yes |
| 297 | SKA3 |  |  | 2985 | -0.592 | -0.3534 | Yes |
| 298 | TPX2 |  |  | 3006 | -0.606 | -0.3573 | Yes |
| 299 | RTKN2 |  |  | 3020 | -0.613 | -0.3588 | Yes |
| 300 | NOVA1 |  |  | 3040 | -0.627 | -0.3623 | Yes |
| 301 | AURKB |  |  | 3056 | -0.639 | -0.3644 | Yes |
| 302 | CLVS2 |  |  | 3059 | -0.642 | -0.3623 | Yes |
| 303 | PHF19 |  |  | 3060 | -0.643 | -0.3596 | Yes |
| 304 | LMNB2 |  |  | 3061 | -0.644 | -0.3568 | Yes |
| 305 | NCAPD3 |  |  | 3064 | -0.646 | -0.3547 | Yes |
| 306 | CDKN1B |  |  | 3065 | -0.646 | -0.3520 | Yes |
| 307 | FANCI |  |  | 3074 | -0.656 | -0.3517 | Yes |
| 308 | C21orf58 |  |  | 3078 | -0.659 | -0.3499 | Yes |
| 309 | GTSE1 |  |  | 3083 | -0.663 | -0.3483 | Yes |
| 310 | TMPO |  |  | 3088 | -0.666 | -0.3468 | Yes |
| 311 | ANP32A |  |  | 3090 | -0.666 | -0.3443 | Yes |
| 312 | SAP30 |  |  | 3096 | -0.671 | -0.3430 | Yes |
| 313 | MAD2L1 |  |  | 3099 | -0.675 | -0.3408 | Yes |
| 314 | RFC3 |  |  | 3108 | -0.683 | -0.3404 | Yes |
| 315 | KIFC1 |  |  | 3110 | -0.684 | -0.3378 | Yes |
| 316 | DACH1 |  |  | 3112 | -0.686 | -0.3352 | Yes |
| 317 | LSM4 |  |  | 3115 | -0.688 | -0.3329 | Yes |
| 318 | PA2G4 |  |  | 3116 | -0.690 | -0.3300 | Yes |
| 319 | DBF4 |  |  | 3117 | -0.690 | -0.3270 | Yes |
| 320 | MYBL1 |  |  | 3119 | -0.691 | -0.3244 | Yes |
| 321 | LMNB1 |  |  | 3121 | -0.692 | -0.3218 | Yes |
| 322 | PRR11 |  |  | 3125 | -0.698 | -0.3198 | Yes |
| 323 | SMC4 |  |  | 3126 | -0.699 | -0.3168 | Yes |
| 324 | CENPK |  |  | 3127 | -0.700 | -0.3138 | Yes |
| 325 | RFC4 |  |  | 3129 | -0.701 | -0.3111 | Yes |
| 326 | NFIC |  |  | 3133 | -0.707 | -0.3091 | Yes |
| 327 | GMNN |  |  | 3140 | -0.719 | -0.3079 | Yes |
| 328 | KIF15 |  |  | 3142 | -0.723 | -0.3052 | Yes |
| 329 | BTG3 |  |  | 3148 | -0.727 | -0.3037 | Yes |
| 330 | CDK1 |  |  | 3149 | -0.727 | -0.3006 | Yes |
| 331 | NDUFAF3 |  |  | 3150 | -0.728 | -0.2975 | Yes |
| 332 | MKI67 |  |  | 3158 | -0.738 | -0.2966 | Yes |
| 333 | ORC6 |  |  | 3159 | -0.738 | -0.2934 | Yes |
| 334 | TK1 |  |  | 3162 | -0.745 | -0.2909 | Yes |
| 335 | DNAJC9 |  |  | 3167 | -0.747 | -0.2890 | Yes |
| 336 | STMN1 |  |  | 3171 | -0.751 | -0.2867 | Yes |
| 337 | MCM8 |  |  | 3178 | -0.758 | -0.2854 | Yes |
| 338 | CUX1 |  |  | 3181 | -0.760 | -0.2828 | Yes |
| 339 | USP1 |  |  | 3183 | -0.762 | -0.2799 | Yes |
| 340 | BRCA1 |  |  | 3185 | -0.765 | -0.2770 | Yes |
| 341 | SORT1 |  |  | 3186 | -0.765 | -0.2737 | Yes |
| 342 | CDCA2 |  |  | 3189 | -0.767 | -0.2711 | Yes |
| 343 | MAGI1 |  |  | 3193 | -0.771 | -0.2687 | Yes |
| 344 | STON2 |  |  | 3196 | -0.776 | -0.2661 | Yes |
| 345 | XRCC2 |  |  | 3197 | -0.777 | -0.2628 | Yes |
| 346 | BARD1 |  |  | 3201 | -0.783 | -0.2604 | Yes |
| 347 | MID1 |  |  | 3207 | -0.792 | -0.2586 | Yes |
| 348 | BIRC5 |  |  | 3208 | -0.793 | -0.2552 | Yes |
| 349 | FBXO5 |  |  | 3212 | -0.797 | -0.2528 | Yes |
| 350 | DIAPH3 |  |  | 3213 | -0.800 | -0.2494 | Yes |
| 351 | RRM1 |  |  | 3215 | -0.801 | -0.2463 | Yes |
| 352 | MPPED2 |  |  | 3216 | -0.803 | -0.2428 | Yes |
| 353 | SPC25 |  |  | 3219 | -0.806 | -0.2400 | Yes |
| 354 | MDK |  |  | 3224 | -0.811 | -0.2379 | Yes |
| 355 | UBE2C |  |  | 3225 | -0.812 | -0.2344 | Yes |
| 356 | SYNE2 |  |  | 3231 | -0.820 | -0.2325 | Yes |
| 357 | NUSAP1 |  |  | 3237 | -0.825 | -0.2306 | Yes |
| 358 | FOXM1 |  |  | 3238 | -0.826 | -0.2271 | Yes |
| 359 | HMGN2 |  |  | 3242 | -0.833 | -0.2245 | Yes |
| 360 | NFIX |  |  | 3246 | -0.841 | -0.2218 | Yes |
| 361 | DTL |  |  | 3249 | -0.848 | -0.2189 | Yes |
| 362 | MYO10 |  |  | 3255 | -0.857 | -0.2168 | Yes |
| 363 | LCORL |  |  | 3256 | -0.859 | -0.2132 | Yes |
| 364 | CCP110 |  |  | 3262 | -0.870 | -0.2111 | Yes |
| 365 | CENPM |  |  | 3269 | -0.881 | -0.2092 | Yes |
| 366 | CHAF1A |  |  | 3270 | -0.882 | -0.2055 | Yes |
| 367 | CDCA5 |  |  | 3273 | -0.885 | -0.2023 | Yes |
| 368 | H2AFX |  |  | 3282 | -0.898 | -0.2011 | Yes |
| 369 | GAS2L3 |  |  | 3283 | -0.899 | -0.1972 | Yes |
| 370 | HELLS |  |  | 3284 | -0.901 | -0.1934 | Yes |
| 371 | LDLRAD3 |  |  | 3285 | -0.904 | -0.1895 | Yes |
| 372 | CDKN3 |  |  | 3293 | -0.916 | -0.1879 | Yes |
| 373 | FRMD4B |  |  | 3294 | -0.917 | -0.1840 | Yes |
| 374 | ZWINT |  |  | 3296 | -0.925 | -0.1803 | Yes |
| 375 | NKAIN3 |  |  | 3301 | -0.933 | -0.1776 | Yes |
| 376 | PLCB1 |  |  | 3302 | -0.934 | -0.1736 | Yes |
| 377 | TMTC4 |  |  | 3303 | -0.936 | -0.1696 | Yes |
| 378 | STK17A |  |  | 3312 | -0.949 | -0.1682 | Yes |
| 379 | FBLN1 |  |  | 3317 | -0.954 | -0.1654 | Yes |
| 380 | PCNA |  |  | 3319 | -0.963 | -0.1616 | Yes |
| 381 | GNG4 |  |  | 3323 | -0.971 | -0.1584 | Yes |
| 382 | UBE2T |  |  | 3330 | -0.979 | -0.1562 | Yes |
| 383 | ROBO2 |  |  | 3337 | -0.998 | -0.1538 | Yes |
| 384 | EZH2 |  |  | 3342 | -1.015 | -0.1508 | Yes |
| 385 | DEK |  |  | 3344 | -1.025 | -0.1467 | Yes |
| 386 | RAD51AP1 |  |  | 3345 | -1.030 | -0.1424 | Yes |
| 387 | PBK |  |  | 3354 | -1.046 | -0.1405 | Yes |
| 388 | MCM10 |  |  | 3356 | -1.050 | -0.1363 | Yes |
| 389 | LRRN1 |  |  | 3359 | -1.060 | -0.1324 | Yes |
| 390 | ESCO2 |  |  | 3367 | -1.088 | -0.1300 | Yes |
| 391 | NCAPG |  |  | 3370 | -1.098 | -0.1260 | Yes |
| 392 | NFIB |  |  | 3388 | -1.166 | -0.1265 | Yes |
| 393 | CHD7 |  |  | 3389 | -1.167 | -0.1215 | Yes |
| 394 | HMGB2 |  |  | 3390 | -1.184 | -0.1165 | Yes |
| 395 | CLSPN |  |  | 3391 | -1.184 | -0.1114 | Yes |
| 396 | CENPU |  |  | 3398 | -1.230 | -0.1081 | Yes |
| 397 | ATAD2 |  |  | 3415 | -1.278 | -0.1078 | Yes |
| 398 | MYEF2 |  |  | 3419 | -1.290 | -0.1032 | Yes |
| 399 | RRM2 |  |  | 3420 | -1.297 | -0.0977 | Yes |
| 400 | CSRP2 |  |  | 3425 | -1.330 | -0.0933 | Yes |
| 401 | HIST1H1C |  |  | 3427 | -1.335 | -0.0879 | Yes |
| 402 | HES6 |  |  | 3431 | -1.366 | -0.0831 | Yes |
| 403 | TYMS |  |  | 3436 | -1.391 | -0.0784 | Yes |
| 404 | FJX1 |  |  | 3440 | -1.441 | -0.0732 | Yes |
| 405 | SLCO5A1 |  |  | 3441 | -1.474 | -0.0669 | Yes |
| 406 | HIST1H1D |  |  | 3466 | -1.743 | -0.0672 | Yes |
| 407 | HIST1H1B |  |  | 3467 | -1.770 | -0.0597 | Yes |
| 408 | LINC00461 |  |  | 3473 | -1.798 | -0.0536 | Yes |
| 409 | INSM1 |  |  | 3483 | -1.929 | -0.0483 | Yes |
| 410 | ASCL1 |  |  | 3484 | -1.937 | -0.0400 | Yes |
| 411 | POU3F2 |  |  | 3487 | -2.092 | -0.0317 | Yes |
| 412 | ID4 |  |  | 3488 | -2.097 | -0.0228 | Yes |
| 413 | NFIA |  |  | 3492 | -2.214 | -0.0143 | Yes |
| 414 | HIST1H4C |  |  | 3493 | -2.248 | -0.0047 | Yes |
| 415 | CKB |  |  | 3497 | -2.376 | 0.0045 | Yes |
Table: GSEA details [plain text format]

  

Fig 2: RICHARDS\_NOWAKOWSKI\_SCIENCE\_2017\_IPC\_DIV1\_UPREG: Random ES distribution      
 Gene set null distribution of ES for **RICHARDS\_NOWAKOWSKI\_SCIENCE\_2017\_IPC\_DIV1\_UPREG**
